# Supplementary material for: STAT3-Induced lncRNA SNHG17 Exerts Oncogenic Effects on Ovarian Cancer through Regulating CDK6
Source: Mol Ther Nucleic Acids. 2020 Aug 8;22:38–49. doi: 10.1016/j.omtn.2020.08.006 (PMC7490451; doi:10.1016/j.omtn.2020.08.006)
Supplement: Document S1. Figures S1 and S2 and Supplemental Materials and Methods [file mmc1.pdf]

**OMTN, Volume *22***

## Supplemental Information

# STAT3-Induced lncRNA SNHG17 Exerts Oncogenic Effects on Ovarian Cancer through Regulating CDK6

**Xuefeng Pan, Zhiheng Guo, Yanyan Chen, Shu Zheng, Min Peng, Yi Yang, and Zhenpeng Wang**

A

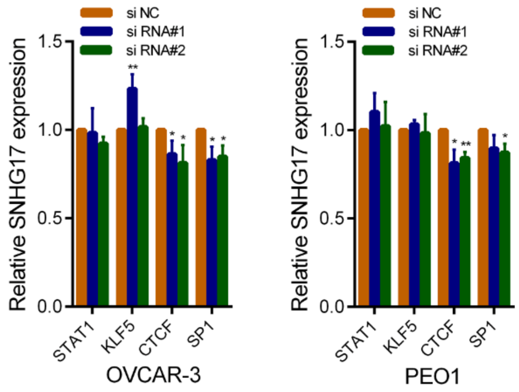


**Figure S1. Explore potential transcription factors affecting SNHG17 transcription. (A)** The SNHG17 expression was detected in OVCAR-3 and PEO1 cells transfected with STAT1, KLF5, CTCF or SP1 siRNAs. *$P < 0.05$ and **$P < 0.01$.

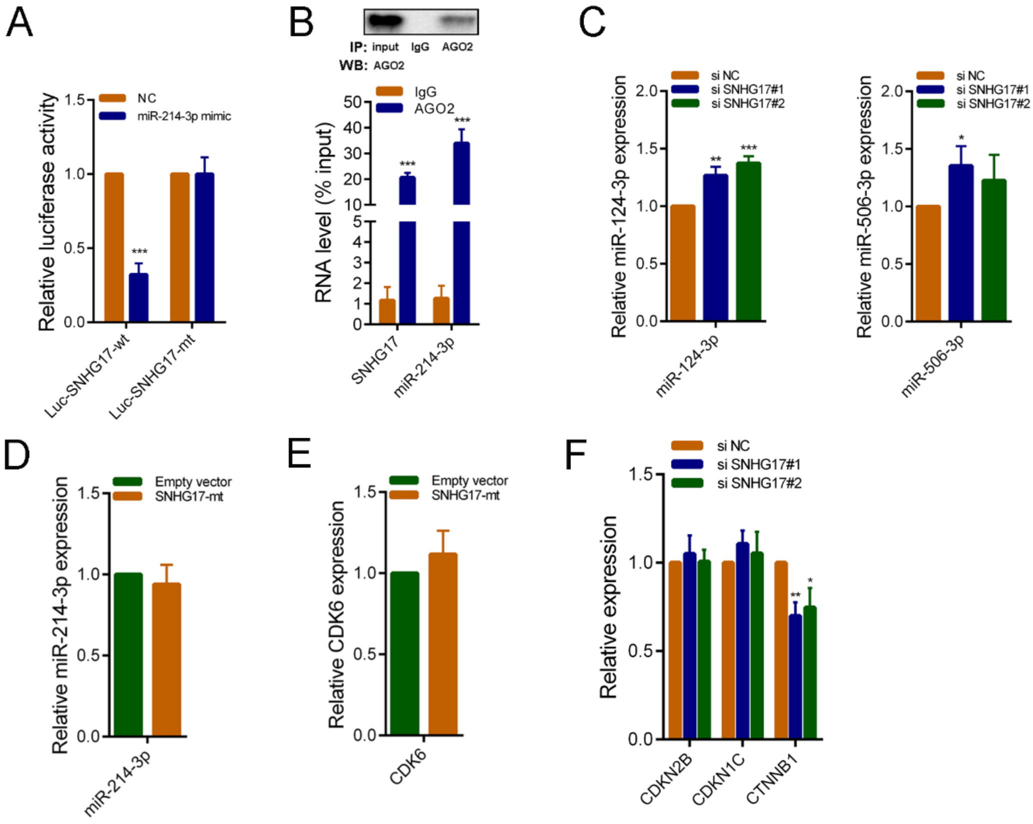


**Figure S2. SNHG17 regulates CDK6 expression through sponging miR-214-3p in OC. (A)** Dual-luciferase reporter assays of wild type (Luc-SNHG17-wt) and mutant type (Luc-SNHG17-mt) luciferase report vectors in PEO1 cells. **(B)** RIP assays with an anti-Ago2 antibody to assess endogenous Ago2 binding RNAs in PEO1 cells. **(C)** MiR-124-3p and miR-506-3p expression was detected in OVCAR-3 cells transfected with SNHG17 siRNAs. **(D)** MiR-214-3p expression was detected in OVCAR-3 cells transfected with SNHG17-mt vectors. **(D)** CDK6 expression was detected in OVCAR-3 cells transfected with SNHG17-mt vectors. **(E)** Expression of CDKN2B, CDKN1C and CTNNB1 was detected by qRT-PCR in OVCAR-3 cells after transfection with SNHG17 siRNAs. *P < 0.05, **P < 0.01 and ***P < 0.001.

## Supplemental Methods

### CCK-8 assay and colony formation assay

For CCK-8 assay, OVCAR-3 and PEO1 cells were seeded into 96-well plates at the density of $1\times10^3$ (cells/well), and the absorbance at 450nm was measured on days 1, 2, 3, 4 and 5 with 10 μl of CCK-8 solution treated. For cell colony formation assays, 24 hours after transfection, 500 OVCAR-3 or PEO1 cells were incubated in 6-well plates at 37˚C, 5% $CO_2$. Two weeks later, the cells were stained with crystal violet (0.2%) for 30 minutes and the colony numbers were counted.

### 5-Ethynyl-20-deoxyuridine (EdU) incorporation assay

OVCAR-3 and PEO1 cells were seeded at a density of $5\times10^3$ cells per well in 96-well plates and cultured overnight. The newly synthesized DNA of the cells was assessed by the EdU incorporation assay using a Cell-Light EdU DNA Cell Proliferation Kit (Ribobio, China), according to the manufacturer's instructions. The EdU incorporation rate was expressed as the ratio of EdU positive cells (red cells) to total Hoechst33342 positive cells (blue cells).

### Flow cytometry

Flow cytometry cell apoptosis was analyzed using the Annexin V-FITC/ (PI) Apoptosis Detection Kit (BD, USA) according to the protocol. OVCAR-3 and PEO1 cells were firstly trypsinized and washed with cold PBS, then they were stained with FITC and PI and then analyzed using FACScan (BD, USA). For cell cycle analysis, OVCAR-3 and PEO1 cells were firstly trypsinized and washed with cold PBS, then they were fixed in 70% ethanol for 24 hours and stained with Propidium Iodide (PI) for 30 minutes. Finally, the cells were analyzed by FACScan flow cytometer (BD, USA). The cell apoptosis data and cell cycle data was analyzed by Flowjo software (Tree Star, USA).

### RNA isolation and quantitative real-time PCR

Total RNA was extracted from tissues and cells using TRIzol reagent (Invitrogen, Carlsbad, CA, USA). Reverse transcription of lncRNA and mRNA was performed using a PrimeScriptTM RT Master Mix Kit (TaKaRa, Osaka, Japan). MicroRNA expression was detected by a Hairpin microRNA quantitation kit (Genepharma, China). Quantitative RT-PCR was performed using a standard protocol from the SYBR Green

PCR kit (Toyobo, Osaka, Japan). The following primer sequences were used for qRT-PCR: for SNHG17, TGCTTGTAAGGCAGGGTCTC (forward) and ACAGCCACTGAAAGCATGTG (reverse); for CDK6, CCAGATGGCTCTAACCTCAGT (forward) and AACTTCCACGAAAAAGAGGCTT (reverse); ; for CDKN2B (P15), ACTAGTGGAGAAGGTGCGACAG (forward) and GCTGCCCATCATCATGACCTG (reverse) ; for CDKN1C (P57), TGAACGCCGAGGACCAGAA (forward) and ACCGAGTCGCTGTCCACTT (reverse) ; for CTNNB1, CCATCTGTGCTCTTCGTCATCT (forward) and GGAATGGCACCCTGCTCAC (reverse); for GAPDH, ATGGGGAAGGTGAAGGTCGG (forward) and GACGGTGCCATGGAATTTGC (reverse).

**Protein extraction and western blot**

Total proteins were extracted from cultured cells using cell lysis buffer. Then, the protein samples were loaded onto 10% sodium dodecyl sulfate polyacrylamide gel electrophoresis. The membranes were blocked with 5 % non-fat milk in Tris-buffered saline and incubated with a specific primary antibody and a secondary antibody. Protein expression was detected by enhanced chemiluminescence kit. Antibodies used in western blot: anti-Cyclin D1(#55506, Cell Signaling Technology, USA), anti-Cyclin D2(#3741, Cell Signaling Technology, USA) anti-CDK6 (#13331, Cell Signaling Technology, USA) and anti-GAPDH (#5174, Cell Signaling Technology, USA).

**Immunohistochemistry**

IHC staining was performed using Dako Envision System (Dako, USA) according to the manufacturer's guidelines. The IHC-stained tissue sections were scored by two pathologists who were blinded to the clinical parameters, respectively. The percentage of immunostaining and the staining intensity (0, negative; 1+, weak; 2+, moderate; and 3+, strong) were recorded. An H-score was calculated using the following formula: [1 × (% cells 1+) + 2 × (% cells 2+) + 3 × (% cells 3+)] × 100. The maximum H-score would be 300, corresponding to 100% of cells with strong intensity. Antibodies used in

IHC: anti-Ki-67 (#9449, Cell Signaling Technology, USA) anti-CDK6 (#13331, Cell Signaling Technology, USA).

**Gene set enrichment analysis**

Gene set enrichment analysis (GSEA) was used to explore the pathways and gene sets associated with SNHG17 in ovarian cancer. Gene expression profiles of 370 ovarian cancer samples were downloaded from TCGA database. According to the SNHG17 expression level, the samples were grouped as the high SNHG17 group and low SNHG17 group respectively. GSEA v3.0 was used to determine whether the members of the gene sets from the MSigDB database were randomly distributed at the top or bottom of the ranking. If most members of a gene set were positively or negatively related to SNHG17, the set was associated with SNHG17 expression.

**Chromatin immunoprecipitation assay**

ChIP assays were performed using the ChIP Assay Kit (Beyotime, China) according to the manual with slight modifications. OVCAR-3 and PEO1 cells were cross-linked with 1% formaldehyde solution for 10 min at room temperature and quenched with 125 mM glycine. DNA fragments ranging from 200 to 500 bp were yielded via sonication. Then the lysates were immunoprecipitated with anti-STAT3 (#12640, Cell Signaling Technology, USA) or normal rabbit IgG antibody. Immunoprecipitated DNAs were analyzed by qRT-PCR and ChIP primers for the promoter region of SNHG17 are: GATTTGTGGGCCATGAGTCC (forward) and GCGTGAGAAGATGAGCCAAG (reverse).

**RNA pull-down assay**

SNHG17 template DNA was transcribed in vitro with Biotin RNA Labeling Mix and T7 RNA polymerase (Roche, Switzerland) and purified with a RNeasy Mini Kit (Qiagen, USA) according to the manufacturer’s instructions. RNA-bound beads were incubated with total cell lysates of OVCAR-3, and eluted RNA was purified and assessed by qRT-PCR.

**RNA immunoprecipitation assay**

The RIP assay was performed using the Magna RNA immunoprecipitation Kit (Millipore, USA) following the the manual. Briefly, OC cells were lysed in RIP lysis

buffer. Magnetic beads were pre-incubated with anti-AGO2 (#ab32381, Abcam, USA) or IgG antibody for 30 minutes at room temperature and the cell lysates was immunoprecipitated with beads for 6 hours at 4°C. After that, RNA was purified and detected by qRT-PCR.
