# Supplementary material for: STAT3-Induced lncRNA SNHG17 Exerts Oncogenic Effects on Ovarian Cancer through Regulating CDK6
Source: Mol Ther Nucleic Acids. 2020 Aug 8;22:38–49. doi: 10.1016/j.omtn.2020.08.006 (PMC7490451; doi:10.1016/j.omtn.2020.08.006)
Supplement: Document S2. Article plus Supplemental Information [file mmc2.pdf]

Molecular Therapy
Nucleic Acids
Original Article

STAT3-Induced lncRNA SNHG17 Exerts Oncogenic Effects on Ovarian Cancer through Regulating CDK6

Xuefeng Pan,[1,5] Zhiheng Guo,[1,5] Yanyan Chen,[2] Shu Zheng,[1] Min Peng,[1] Yi Yang,[3] and Zhenpeng Wang[4]

[1]Department of Obstetrics, The First Hospital of Jilin University, Changchun 130021, Jilin, China; [2]Department of Ophthalmology, The First Hospital of Jilin University, Changchun 130021, Jilin, China; [3]Center for Reproductive Medicine and Center of Prenatal Diagnosis, The First Hospital of Jilin University, Changchun 130021, Jilin, China; [4]Department of Gynecologic Oncology, The First Hospital of Jilin University, Changchun 130021, Jilin, China

Emerging studies indicate that long noncoding RNAs (lncRNAs) play crucial roles in ovarian cancer (OC). By analyzing high-throughput data, we found that SNHG17 was highly expressed in multiple OC cohorts. However, its functions in OC were not explored. In this study, lncRNA expression in OC was analyzed by a series of microarray data. The functions of SNHG17 were investigated by various *in vitro* and *in vivo* assays. Fluorescence *in situ* hybridization (FISH), RNA pull-down, chromatin immunoprecipitation (ChIP), RNA immunoprecipitation (RIP), and luciferase reporter assays were used to reveal the potential mechanisms involved in the effects of SNHG17. We found that SNHG17 was overexpressed in OC and that the oncogenic transcription factor STAT3 was involved in promoting its expression. In addition, high SNHG17 expression was associated with a poor prognosis in OC. Functional analysis revealed that SNHG17 could promote OC cell growth. Mechanistically, SNHG17 was found to be located predominantly in the cytoplasm. It could regulate expression of CDK6, an important cell-cycle regulator, by acting as a molecular sponge for miR-214-3p. In summary, our study suggested that SNHG17 acted as an oncogene in OC, which might serve as a novel target for OC diagnosis and therapy.

INTRODUCTION

Ovarian cancer (OC) is one of the most common gynecologic cancers. Its morbidity ranks seventh among all malignant cancers in women, and it is the fifth leading cause of tumor-related deaths in women.[1,2] Due to improvements in diagnosis and treatment techniques, the overall prognosis for OC patients has improved. However, drug resistance, extensive metastases, and relapse often occur in advanced OC, which leads to a 5-year survival rate in OC of less than 40%.[3–5] Thus, further exploration of the molecular mechanism of OC development and progression is urgently needed.

Long noncoding RNAs (lncRNAs) are a class of RNAs longer than 200 nucleotides (nt) in length with little or no protein-coding capacity.[6] These transcripts were previously thought to be transcriptional "noise," but as their study has progressed, some of them have been found to play important roles in a variety of physiological or pathological processes.[7–9] Recent studies have revealed that some lncRNAs are abnormally expressed and play vital roles in OC, such as HOST2, MALAT1, PVT1, and PTAR.[10–13] However, the roles of most lncRNAs in OC remain largely unknown, and further studies are needed.

Small nucleolar RNA host gene 17 (SNHG17) is located on 20q11. It has recently been reported to act as an oncogene in various tumors, such as colorectal cancer, gastric cancer, and non-small-cell lung cancer.[14–16] Through analyzing the microarray data of OC tissues, we found that it was also widely overexpressed in OC.

In this study, we explored the expression pattern, clinical significance, functions, and molecular mechanisms of SNHG17 in OC. SNHG17 was highly expressed in OC, and its high expression was associated with a poor prognosis in OC patients. In addition, we demonstrated that the high SNHG17 expression was due to STAT3 activation. Silencing SNHG17 inhibited OC cell growth and induced cell-cycle arrest. SNHG17 is primarily located in the cytoplasm and can increase CDK6 expression by acting as a sponge for miR-214-3p. Taken together, the results of our study revealed the effects of SNHG17 in OC and provide a novel theoretical target for the diagnosis and treatment of OC.

RESULTS

SNHG17 Expression Is Upregulated in OC, and Its High Expression Correlates with a Poor Prognosis

First, we analyzed the microarray data of OC tissues in the GEO database (GEO: GSE54388, GSE38666, and GSE 14407) and identified the

Received 18 March 2020; accepted 7 August 2020;
https://doi.org/10.1016/j.omtn.2020.08.006.

[5]These authors have contributed equally to this work.

Correspondence: Zhenpeng Wang, Department of Gynecologic Oncology, The First Hospital of Jilin University, Changchun 130021, Jilin, China.
E-mail: wangzhenpeng1987@163.com
Correspondence: Yi Yang, Center for Reproductive Medicine and Center of Prenatal Diagnosis, The First Hospital of Jilin University, Changchun 130021, Jilin, China.
E-mail: yangyi3009@126.com

 © 2020 The Author(s).
This is an open access article under the CC BY license (http://creativecommons.org/licenses/by/4.0/).

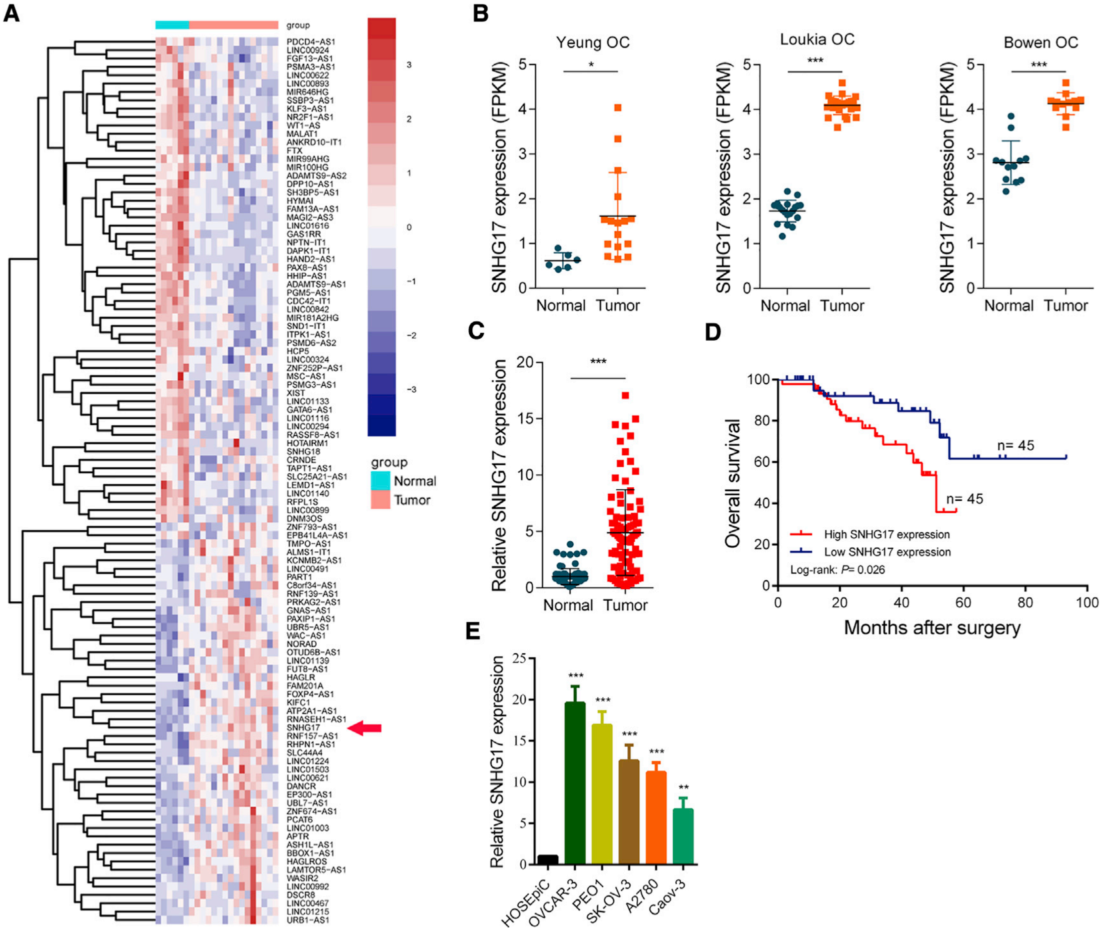

Figure 1. SNHG17 Is Upregulated in OC, and a High SNHG17 Expression Predicts a Poor Prognosis

(A) Heatmap of aberrantly expressed lncRNAs in OC (GEO: GSE54388). Red in the heatmap indicates upregulation, and blue indicates downregulation. The red arrow denotes SNHG17. (B) The expression of SNHG17 in the Yeung OC cohort (GEO: GSE54388), Loukia OC cohort (GEO: GSE38666), and Bowen OC cohort (GEO: GSE14407). (C) qRT-PCR analysis of SNHG17 expression in 90 pairs of OC and corresponding adjacent normal tissues. (D) Kaplan-Meier analysis of OC patients' overall survival based on SNHG17 expression (n = 90; p = 0.026); a tissue sample whose threshold cycle (CT) value of SNHG17 minus a CT value of GAPDH less than −6.46 was defined as high SNHG17 expression. (E) qRT-PCR analysis of SNHG17 expression in OC cell lines (OVCAR-3, PEO1, SK-OV-3, A2780, and Caov-3) and normal human ovarian surface epithelial cells (HOSEpiCs). $^{*}p < 0.05$; $^{**}p < 0.01$; $^{***}p < 0.001$.

abnormally expressed lncRNAs in OC (Figure 1A). We found that SNHG17 was significantly highly expressed in OC tissues compared to adjacent tissues in all these OC cohorts (Figure 1B). We also measured the expression of SNHG17 in 90 paired OC and adjacent tissues by qRT-PCR; consistent with the microarray results, SNHG17 expression in tumor tissues was significantly increased (Figure 1C). To explore the clinical significance of SNHG17 in OC, we divided 90 patients into two groups according to SNHG17 expression. The statistical analysis results demonstrated that SNHG17 expression was correlated with the International Federation of Gynecology and Obstetrics (FIGO) stage ($p < 0.001$), histological grade ($p = 0.015$), and tumor size stage ($p = 0.010$; Table 1). Moreover, we examined the prognostic value of SNHG17 in OC. The survival analysis results showed that those OC patients with a high SNHG17 expression had a significantly worse prognosis (Figure 1D). In addition, we examined the expression of SNHG17 in OC cell lines and normal human ovarian surface epithelial cells (HOSEpiCs). The results indicated that its expression was significantly higher in OC cells than in the normal ovarian epithelial cells (Figure 1E).

STAT3 Activates the Transcription of SNHG17 in OC

We next explored potential factors that induced the high expression of SNHG17 in OC. We used the JASPAR[17] tool to predict transcription factors that might bind to the promoter region of SNHG17, and the oncogenic transcriptional factor STAT3 achieved the highest score. We silenced STAT3 in OVCAR-3 cells and PEO1 cells and found that the expression of SNHG17 was decreased. Conversely, the overexpression of STAT3 caused an increase in SNHG17 expression (Figure 2A). We also detected that several other transcription factors got high scores (STAT1, KLF5, CTCF, and SP1), but silencing

Table 1. The Clinical Characters of 90 OC Patients

Characteristics	Number of Cases	SNHG17 Expression Low (n = 45)	SNHG17 Expression High (n = 45)	p[a]
Age (years)				
<50	35	17	18	0.829
≥50	55	28	27	
FIGO Stage				
I + II	36	30	6	*<0.001*
III + III	54	15	39	
Lymph Node Metastasis				
Negative	63	35	28	0.107
Positive	27	10	17	
Histological Grade				
High	59	24	35	*0.015*
Low	31	21	10	
CA125 in Serum (U/mL)				
<600	32	17	15	0.660
≥600	58	28	30	
Tumor Size (cm)				
≤5	52	32	20	*0.010*
>5	38	13	25	

[a]Statistically significant results appear in italics.

them had little effect on SNHG17 expression (Figure S1A). Importantly, the chromatin immunoprecipitation (ChIP) assay results revealed that STAT3 could directly bind to the promoter region of SNHG17 (Figure 2B). In addition, STAT3 expression was positively correlated with the expression of SNHG17 in OC tissues (Figure 2C). To further determine the binding region of STAT3 in the SNHG17 promoter, we performed a luciferase reporter assay. The results indicated that STAT3 was mainly bound to the E2 region (−2,348 to −2,338 bp, TTTCTGGGAAG), rather than to the E1 region (−328 to −318 bp, TTTCTGAGAAA; Figure 2D).

SNHG17 Promotes OC Cell Growth *In Vitro* and *In Vivo*

Colony formation assays and cell counting Kit-8 (CCK-8) assays showed that silencing SNHG17 reduced the growth ability of OVCAR-3 and PEO1 cells (Figures 3A and 3B). The results of the 5-ethynyl-20-deoxyuridine (EdU) experiments indicated that the proliferative rates of OC cells decreased significantly after the knockdown of SNHG17 (Figure 3C). Similarly, flow cytometry cell-cycle assays showed that, after SNHG17 silencing, the proportion of cells in the "S" phase decreased and cell-cycle arrest was observed (Figure 3D). In addition, cell apoptosis rates were detected by flow cytometry, and the results showed that the apoptosis rates of OVCAR-3 and PEO1 cells were significantly increased when SNHG17 was silenced (Figure 3E). Next, we examined the effect of SNHG17 on OC cells *in vivo*. We constructed SNHG17 knockdown (shSNHG17) and overexpressing (SNHG17) stably transfected OVCAR-3 cell lines. As shown in Figure 4A, SNHG17 was effectively downregulated in shSNHG17-transfected cells and significantly upregulated in SNHG17-overexpressing cells. The results of the nude mouse xenograft model showed that the tumor volumes in the SNHG17 knockdown group were substantially smaller than those in the negative control (NC) group. Conversely, the tumors formed by the SNHG17-overexpressing cells had significantly larger volumes than those of the control group (Figure 4B). After 4 weeks, the mice were sacrificed, and the tumors were formalin-fixed. The immunohistochemistry (IHC) results of these xenografts demonstrated that the positive rate of Ki-67 in the SNHG17 knockdown group was lower than that in the control group. The positive rate of Ki-67 was higher in the SNHG17-overexpressing group than that in the control group (Figure 4C). In addition, we detected levels of the proliferation-associated proteins cyclin D1 and cyclin D2 by western blot in the SNHG17 small interfering RNA (siRNA)-transfected OC cells. The results demonstrated that both these proteins were significantly decreased (Figure 4D). Gene set enrichment analysis (GSEA) also revealed significant relations between the cell-cycle-related pathways and SNHG17 expression in OC (Figure 4E). To summarize, these results indicate that SNHG17 promotes OC tumor growth both *in vitro* and *in vivo* and that SNHG17 may play a vital role in cell-cycle regulation in OC.

SNHG17 Acts as a Competitive Endogenous RNA (ceRNA) to Sponge miR-214-3p in OC

Considering that the way in which lncRNA works is related to its subcellular localization, we then explored its distribution in OC cells. RNA fluorescence *in situ* hybridization (FISH) experiments showed that SNHG17 was mainly located in the cytoplasm of OVCAR-3 and PEO1 cells (Figure 5A). The results of subcellular fractionation assays also indicated that SNHG17 was primarily distributed in the cytoplasm (Figure 5B). Thus, we hypothesized that SNHG17 might play a role as a ceRNA. We then used the miRDB[18,19] tools to search for microRNAs that could theoretically interact with SNHG17. A set of microRNAs (miR-214-3p, miR-487a-5p, miR-1205, miR-487b-5p, miR-187-5p, miR-761, miR-539-5p, miR-342-5p, and miR-632) achieved relatively high scores. We next conducted RNA pull-down experiments with biotin-labeled SNHG17 in OVCAR-3 cells. As shown in Figure 5C, miR-214-3p, miR-487a-5p, miR-1205, and miR-487b-5p could be pulled down by SNHG17. Compared with other microRNAs, SNHG17 combined the largest amount of miR-214-3p, and it has two binding sites for miR-214-3p. Thus, we chose miR-214-3p for the next study. To test our hypothesis, we examined the expression of miR-214-3p in OVCAR-3 cells when SNHG17 was silenced or overexpressed. As shown in Figure 5D, the expression of miR-214-3p was significantly elevated after SNHG17 was knocked down and was significantly decreased when SNHG17 was overexpressed. We also evaluated the expression of miR-214-3p in OC tissues. In contrast to SNHG17, it was significantly lower in tumor tissues compared to normal tissues (Figure 5E). In addition, we constructed a wild-type (WT) luciferase reporter plasmid (Luc-SNHG17-WT) and a mutant luciferase reporter plasmid (Luc-SNHG17-mt) based on the predicted miR-214-3p and SNHG17 binding sites. The

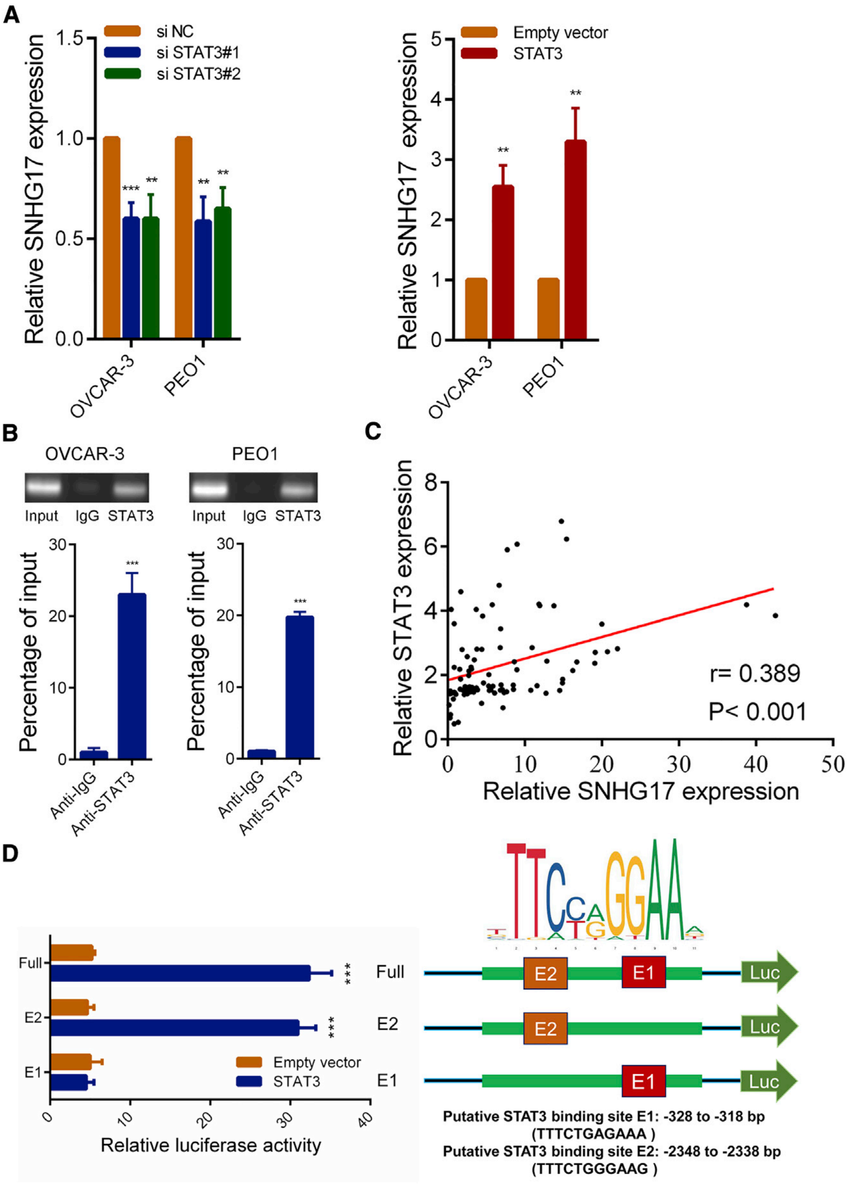

Figure 2. STAT3 Induces a High SNHG17 Expression in OC

(A) SNHG17 expression was detected in OVCAR-3 and PEO1 cells transfected with STAT3 siRNAs or the STAT3-overexpressing vector by qRT-PCR. (B) ChIP assays were conducted to identify STAT3 occupancy in the SNHG17 promoter region. (C) The correlation between STAT3 and SNHG17 expression analyzed in 90 pairs OC samples (n = 90; r = 0.389, p < 0.001). (D) Luciferase reporter assays were used to determine the STAT3 binding sites on the SNHG17 promoter region. **p < 0.01; ***p < 0.001.

results of the luciferase reporter assay indicated that miR-214-3p could bind to Luc-SNHG17-WT but not to Luc-SNHG17-mt (Figure 5F; Figure S2A). In addition, RNA immunoprecipitation (RIP) assays with an AGO2 antibody were performed, and the results revealed that both miR-214-3p and SNHG17 could directly interact with the endogenous AGO2 protein in OVCAR-3 and PEO1 cells (Figure 5G; Figure S2B). In addition, a correlation analysis demonstrated that the expression of miR-214-3p and SNHG17 was negatively correlated in OC tissues (Figure 5H; Figure S2B). It has been reported that SNHG17 can act as a ceRNA to sponge miR-124-3p and miR-506-3p.[20,21] In OVCAR-3 cells, we found that silencing SNHG17 caused a slight increase in miR-124-3p (about 30%), which was much lower than that in miR-214-3p (about 300%), and had almost no effect on the expression of miR-506-3p (Figure S2C). These results indicate that SNHG17 can regulate miR-214-3p expression through working as a ceRNA in OC.

SNHG17 Can Regulate CDK6 Expression by Sponging of miR-214-3p in OC

As is well known, microRNAs exert their functions by inhibiting target genes. We used three tools (PITA,[22] miRmap,[23] and TargetScan[24]) to search for targets of miR-214-3p. CDK6 was identified as a potential target of miR-214-3p by all these tools. Considering that SNHG17 could affect the proliferation of OC cells, we speculated that SNHG17 might exert its effects by absorbing miR-214-3p, thus regulating CDK6 expression. We then constructed the WT luciferase reporter plasmid (WT CDK6-3′ UTR) and the mutant luciferase reporter plasmid (mt CDK6-3′ UTR) based on the predicted binding sites. The relative luciferase activity was significantly decreased in cells cotransfected with the WT CDK6-3′ UTR luciferase reporter and miR-214-3p mimics. However, no significant changes were observed in the mt CDK6-3′ UTR group (Figure 6A). We next studied the effect of SNHG17 on CDK6 expression. The results indicated that when SNHG17 was overexpressed, the expression of CDK6 was elevated, and when both SNHG17 and miR-214-3p was overexpressed, the increase in CDK6 was abolished (Figure 6B). In addition, SNHG17 knockdown could reduce CDK6 expression in OVCAR-3 cells but reduced CDK6 could be rescued when SNHG17 siRNAs and miR-214-3p inhibitors were cotransfected (Figure 6C). The changes in protein levels were consistent with the changes in the RNA levels (Figure 6D). To further verify that SNHG17 could regulate CDK6 expression by sponging miR-214-3p, we constructed a SNHG17-mt vector, which contains mutations at the putative miR-214-3p binding sites (5′-654TGTCCTGCTG T665-3′ to 5′-654ACTGGACGACA665-3′ to 5′-903TCCCTGCTG 912-3′ to 5′-903ACGGACGAC912-3′). As expected, transfection with SNHG17-mt did not changed miR-214-3p and CDK6 expression (Figures S2D and S2E). Besides, we analyzed the expression of CDK6 in 90 paired OC tissues. We found that CDK6 expression

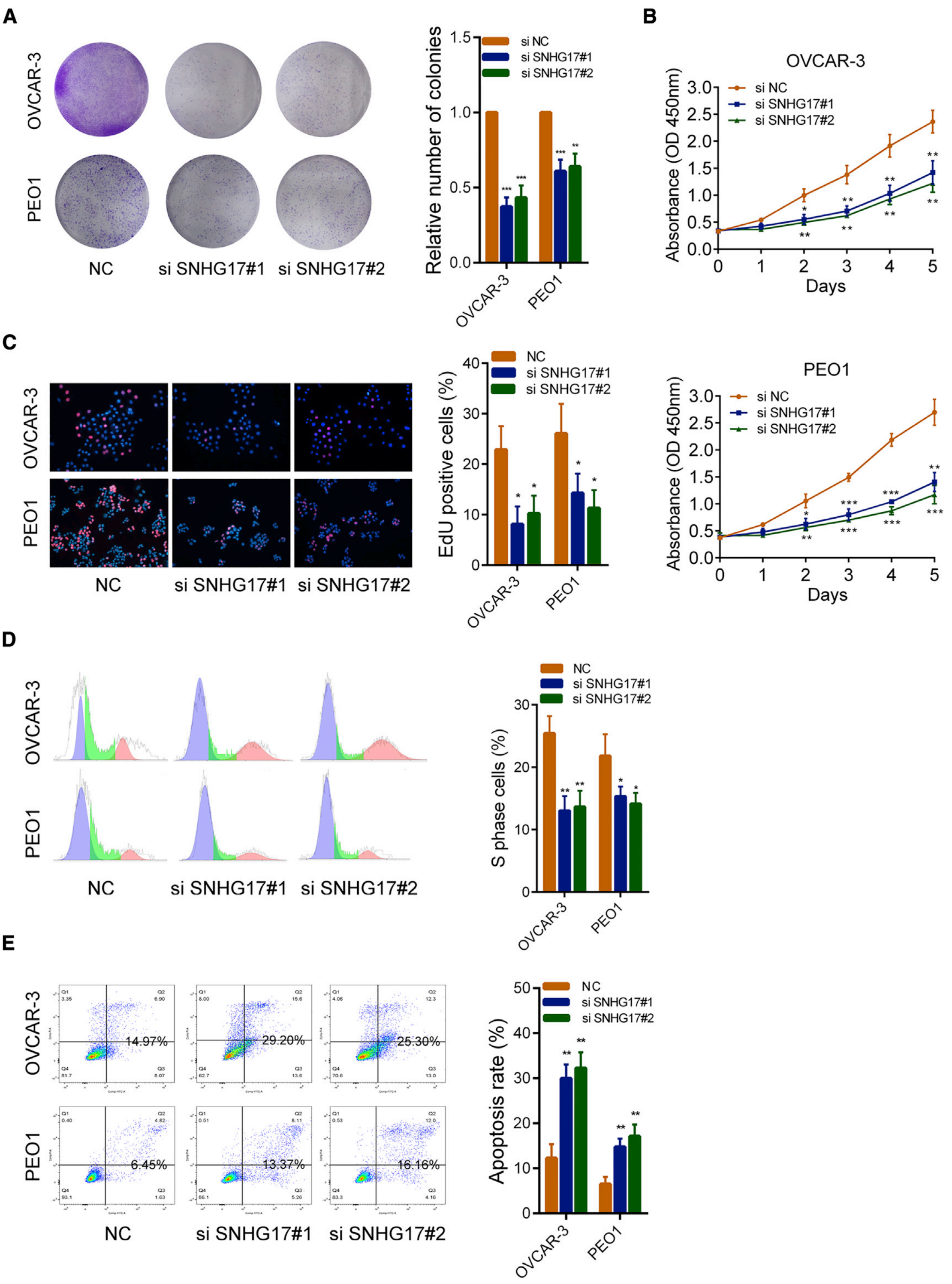

Figure 3. SNHG17 Promotes Proliferation and Inhibits Apoptosis of OC Cells *In Vitro*

(A) Colony formation assays in OVCAR-3 and PEO1 cells transfected with SNHG17 siRNAs. (B) CCK-8 assays in OVCAR-3 and PEO1 cells transfected with SNHG17 siRNAs. (C) EdU assays were used to assess the proliferation ability of OVCAR-3 and PEO1 cells transfected with SNHG17 siRNAs. (D) Flow cytometry cell-cycle assays used to assess the effect of SNHG17 knockdown on cell cycle. (E) Flow cytometry cell apoptosis assays used to assess the effect of SNHG17 silencing on cell apoptosis. *p < 0.05; **p < 0.01; ***p < 0.001.

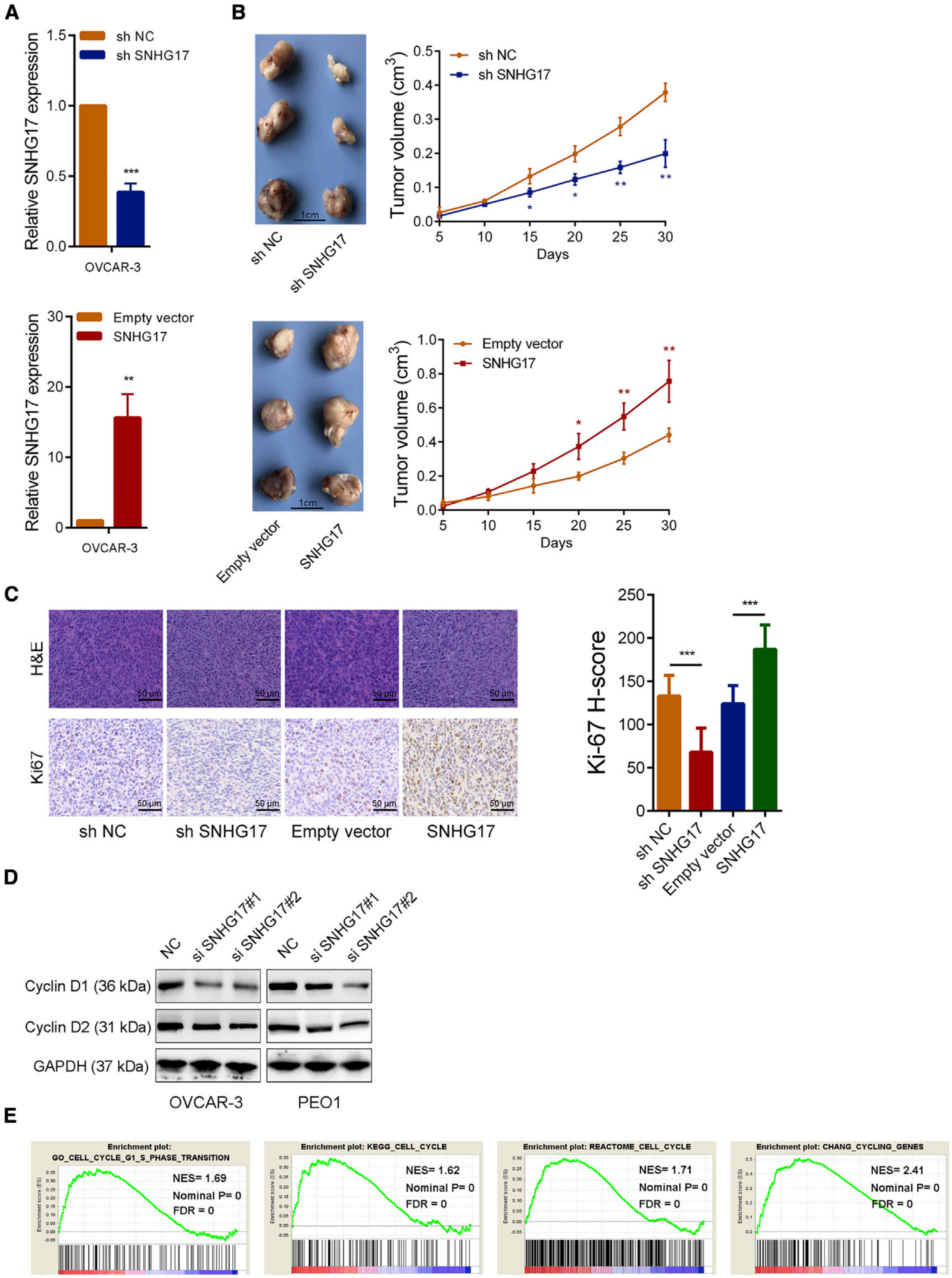

Figure 4. SNHG17 Promotes OC Growth *In Vivo* and Affects Proliferation-Related Pathways

(A) The SNHG17 expression in corresponding stably transfected OVCAR-3 cells was detected by qRT-PCR. (B) Representative images of tumors formed in nude mice from the NC group, shSNHG17 group, and SNHG17-overexpression groups and the tumor volume growth curves after injections in the different groups. (C) Left panel:

(legend continued on next page)

was significantly higher in the cancer tissues compared to the adjacent normal tissues (Figure 6E). Correlation analysis revealed that CDK6 mRNA expression was positively correlated with SNHG17 expression but negatively correlated with miR-214-3p expression (Figures 6F and 6G). In addition, previous studies have shown that SNHG17 can regulate the expression of CDKN2B, CDKN1C, and CTNNB1.[15,21] We found that SNHG17 knockdown had no effect on the expression of CDKN2B and CDKN1C, but it downregulated CTNNB1 in OVCAR-3 cells (Figure S2F). The reasons for this difference need to be further explored.

SNHG17 Promotes OC Cell Growth by Increasing CDK6 Expression

We further determined whether SNHG17 promoted OC cell growth in a CDK6-dependent manner. The CCK-8 experiments showed that overexpression of SNHG17 accelerated the growth rate of OVCAR-3 cells. In contrast, the overexpression of miR-214-3p inhibited the growth rate of OVCAR-3 cells. When the SNHG17 overexpression plasmids and miR-214-3p mimics were cotransfected, the growth acceleration caused by SNHG17 was weakened (Figure 7A, left panel). In addition, silencing CDK6 inhibited the growth of OVCAR-3 cells and could also abolish growth acceleration caused by the overexpression of SNHG17 (Figure 7A, right panel). Consistent with the results of the CCK-8 assays, the EdU experiments revealed that overexpression of SNHG17 could increase the proliferation rate of OVCAR-3. If miR-214-3p was overexpressed or CDK6 was silenced at the same time as the overexpression of SNHG17, it significantly impaired the increase in proliferation ability of OVCAR-3 cells (Figure 7B). In addition, the IHC results indicated that CDK6 expression increased in tumor-bearing tissues formed by SNHG17-overexpressing OVCAR-3 cells and decreased in tumor-bearing tissues formed by the SNHG17-knockdown OVCAR-3 cells (Figure 7C). The results of the western blotting showed that the overexpression of SNHG17 led to CDK6 upregulation and that the protein levels of cyclin D1 and cyclin D2, which were associated with cell proliferation, were also significantly upregulated, while CDK6 knockdown attenuated the effects of SNHG17 on their expression (Figure 7D). In summary, these results suggested that SNHG17 increases the proliferation ability of OC cells through the upregulation of CDK6.

DISCUSSION

OC is a leading cause of cancer incidence and mortality worldwide.[25] The etiology of this lethal disease is not completely understood. Recent studies have shown that some lncRNAs are involved in the development of OC. For example, Wu et al.[26] found that lncRNA ABHD11-AS1 acted as an oncogene in OC by stabilizing RhoC. Wang et al.[27] revealed that lncRNA PANDAR could dictate OC chemoresistance by regulating SFRS2-mediated p53 phosphorylation. Martini et al.[28] demonstrated that lncRNA PVT1 could be a prognostic biomarker in stage I epithelial OC. These studies indicate that a comprehensive understanding of the functions of lncRNA in OC may help us to develop novel diagnosis and treatment strategies. In this study, we determined the expression profiles of abnormally expressed lncRNAs in OC tissues by analyzing high-throughput data. From these aberrantly expressed lncRNAs, we selected SNHG17 for further study. We found that SNHG17 was upregulated in OC tissues and cells and that its high expression predicted a poor prognosis for OC. STAT3, an important cancer-promoting transcription factor, was identified to activate SNHG17 transcription in OC. Functional experiments revealed that SNHG17 could promote OC cell growth both *in vitro* and *in vivo*. Mechanistically, SNHG17 was mainly distributed in the cytoplasm and could upregulate CDK6 as a molecular sponge of miR-214-3p.

Several studies have shown that SNHG17 plays vital roles in various types of disease. Mohamadi et al.[29] reported that a decreased expression of SNHG17 is possibly associated with the development of type 2 diabetes mellitus. Xu et al.[16] found that SNHG17 could promote non-small-cell lung cancer proliferation and migration by regulating the expression of FOXA1, XAF1, and BIK. Chen et al.[30] revealed that SNHG17 could act as a prognosis biomarker of gastric cancer. In addition, Zhang et al.[15] reported that SNHG17 could promote gastric cancer progression through the interaction with PRC2. Here, we first uncovered how SNHG17 exerts oncogenic functions through a new mechanism in OC.

Many studies have revealed that the constitutive activation of STAT3 is common in human cancers. Aberrant STAT3 signaling has been identified to be involved in the initiation and progression of various tumors.[31] Some important anti-apoptotic genes, such as Bcl-XL, Bcl-2, and Mcl-1, have proved to be target genes of STAT3. In addition, proteins related to cell-cycle progression, such as cyclin D1 and c-Myc, were also targets of STAT3.[32,33] Our research indicated that STAT3 could activate SNHG17 expression and that SNHG17 could promote the proliferation of OC cells by regulating CDK6. This may provide a new theory for STAT3 in promoting tumor cell growth. As an important cell-cyclin-dependent kinase, CDK6 can form complexes with D-type cyclins and then inactivate retinoblastoma protein Rb by phosphorylating it. Rb phosphorylation causes E2F family transcription factor release, thereby promoting cell proliferation.[34] CDK6 expression is regulated by many factors.[35] Some studies have reported that CDK6 is a target gene for several microRNAs.[36–38] In this study, we provided evidence that CDK6 was a target of miR-214-3p in OC.

In summary, our results revealed that SNHG17 was upregulated and acted as an oncogene in OC. Its high expression was due to STAT3

representative images for H&E stains and Ki67 immunostaining of tumor samples from the different groups. Right panel: Ki67's H-score statistics result. (D) The cell proliferation-related proteins cyclin D1 and cyclin D2 were detected by western blot after SNHG17 knockdown. (E) The GSEA results were plotted to visualize the correlation between the expression of SNHG17 and the genes related to cell proliferation (GO_CELL_CYCLE_G1_S_PHASE_TRANSITION, KEGG_CELL_CYCLE, REACTOME_CELL_CYCLE, and CHANG_CYCLING_GENES). *p < 0.05; **p < 0.01; ***p < 0.001.

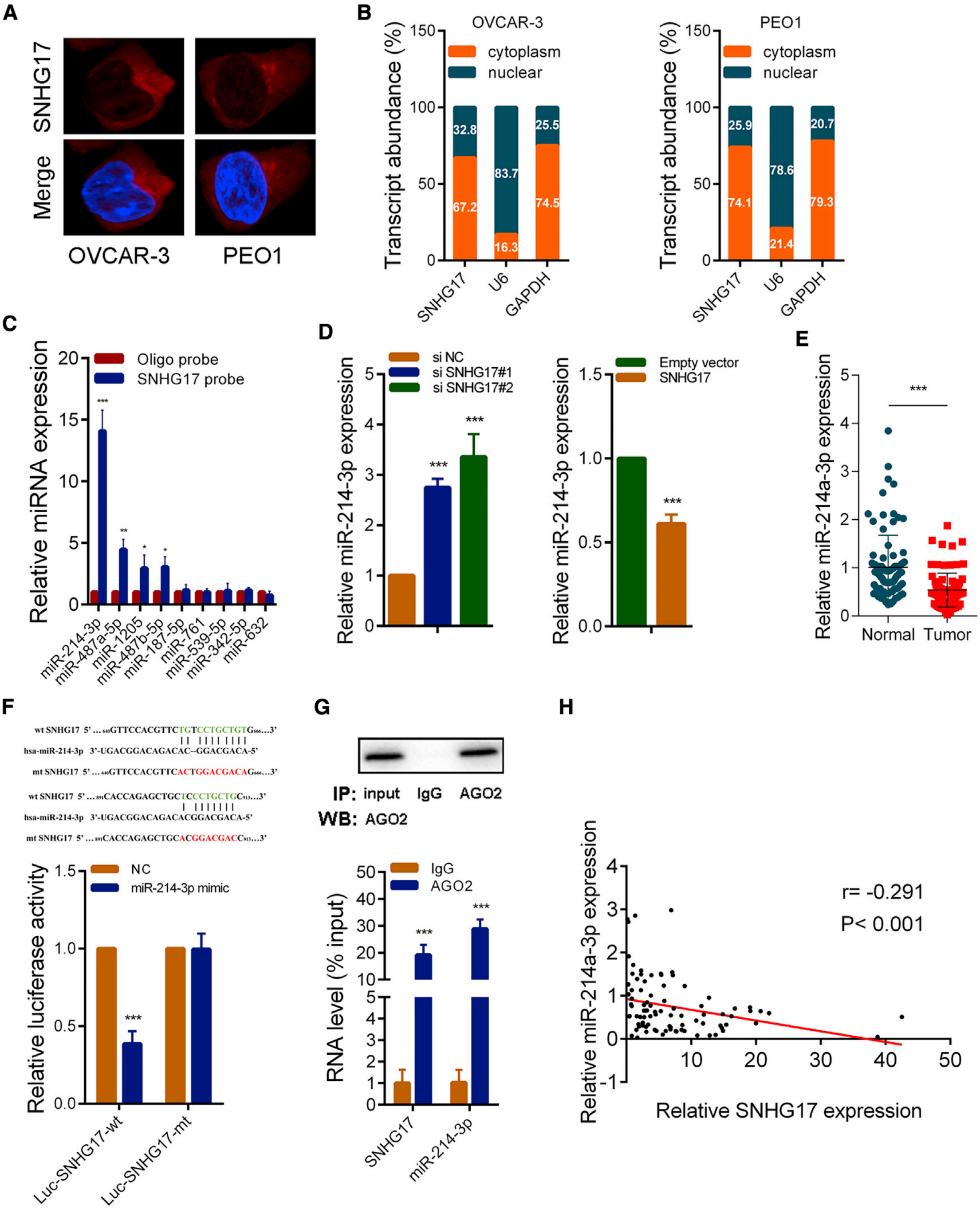

Figure 5. SNHG17 Directly Binds to miR-214-3p and Acts as a Molecular Sponge in the Cytoplasm

(A) Representative FISH images show the distribution of SNHG17 in OVCAR-3 and PEO1 cells (red). The nuclei were stained by DAPI (blue). (B) Relative SNHG17 expression levels in the nuclear and cytoplasm fractions of OVCAR-3 and PEO1 cells. Nuclear controls, U6; cytosolic controls, GAPDH. (C) The relative expression of candidate microRNAs that could potentially bind to SNHG17 was quantified by qRT-PCR after the biotinylated-SNHG17 pull-down assays in OVCAR-3 cells. (D) miR-214-3p expression was detected in OVCAR-3 cells transfected with SNHG17 siRNAs or the SNHG17-overexpressing vector by qRT-PCR. (E) qRT-PCR analysis of miR-214-3p expression in 90 pairs of OC and corresponding adjacent normal tissues. (F) Dual-luciferase reporter assays of WT and mutant type (putative binding sites for miR-214-3p were mutated) SNHG17 luciferase report vectors. Lower panel: sequence alignment of miR-214-3p and their predicted binding sites (green) for SNHG17. Predicted

(legend continued on next page)

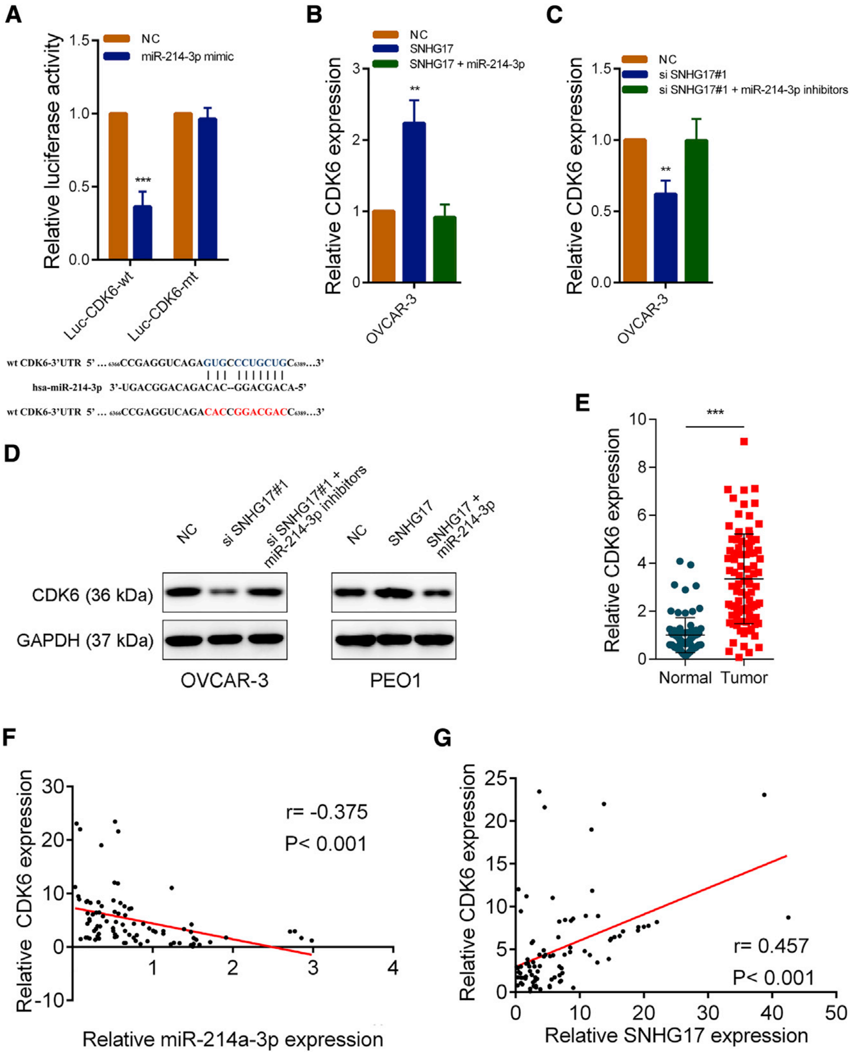

Figure 6. SNHG17 Regulates CDK6 Expression by Acting as a Sponge for miR-214-3p in OC

(A) Dual-luciferase reporter assays with WT and mutant type (putative binding sites for miR-214-3p were mutated) luciferase report vectors of CDK6 3′ UTR. Lower panel: predicted microRNA target sequence (6,377 bp–6,388 bp, blue) in CDK6 3′ UTR (Luc-CDK6-WT) and the positions of mutated nucleotides (red) in CDK6 3′ UTR (Luc-CDK6-mt). (B–D) CDK6 expression was detected by qRT-PCR or western blot in OVCAR-3 cells with the indicated treatment. (E) qRT-PCR analysis of CDK6 expression in 90 pairs of OC and corresponding adjacent normal tissues. (F) The correlation between CDK6 and miR-214-3p in 90 paired OC samples ($n = 90$; $r = -0.375$, $p < 0.001$). (G) The correlation between CDK6 and SNHG17 in 90 paired OC samples ($n = 90$; $r = 0.457$, $p < 0.001$). $^{**}p < 0.01$; $^{***}p < 0.001$.

activation and associated with a poor prognosis in OC. It could function as a molecular sponge for miR-214-3p to weaken the suppressive effect of miR-214-3p on CDK6, thus facilitating the cell-cycle progression of OC cells. Our data suggest that SNHG17 might be a promising prognostic biomarker and therapeutic target for OC.

MATERIALS AND METHODS

Tissue Samples and Clinical Data Collection

Ninety pairs of OC tissues and matched paracancerous tissues were collected from patients who received radical surgery at The First Hospital of Jilin University between May 2012 and July 2014. All samples were stored in a liquid nitrogen container until RNA extraction. Written informed consents were obtained from all these patients, and this study was approved by the Ethics Committee on Human Research of Jilin University. The clinical characteristics of these patients are listed in Table 1.

Cell Culture

The human OC cell lines (OVCAR-3, PEO1, SK-OV-3, A2780, and Caov-3) and normal HOSEpiCs were purchased from the ATCC. These cells were maintained in RPMI 1640 medium (GIBCO, Gaithersburg, MD, USA) with 10% fetal bovine serum, 100 U/mL penicillin, and 0.1 mg/mL streptomycin and were incubated at 37°C in a humidified atmosphere with 5% CO_2.

Plasmid Construction and Cell Transfection

The full-length cDNAs of human STAT3, SNHG17, and SNHG17-mt (mutations at the putative miR-214-3p binding sites) were synthesized and cloned into the expression vector pcDNA3.1. The small hairpin RNA of SNHG17 (shSNHG17) (5′-GAUUGUCAGCUGACCUCUGUCCUGU-3′) was synthesized and cloned into the pGLVH1/GFP/Puro vector (GenePharma, Shanghai, China). STAT3, SNHG17, and CDK6 siRNAs were purchased from Ambion (Waltham, MA, USA). STAT1, KLF5, CTCF, and SP1 siRNAs were purchased from GenePharma (Shanghai, China). Both miR-214-3p mimics and inhibitors were synthesized by RiboBio (Guangzhou, China). The plasmid vectors and siRNAs were transfected into OC cells using Lipofectamine 3000 (Invitrogen, Carlsbad, CA, USA) according to the manufacturer's protocol. For stably transfected cell line construction, stable clones of SNHG17- and

microRNA target sequence (654 bp–665 bp and 902 bp–911 bp, blue) in SNHG17 (Luc-SNHG17-WT) and positions of the mutated nucleotides (red) in SNHG17 (Luc-SNHG17-mt). (G) RIP assays with an anti-Ago2 antibody to assess endogenous Ago2-binding RNAs; immunoglobulin G (IgG) was used as the NC. The levels of SNHG17 and miR-214-3p were determined by qRT-PCR and presented as the fold enrichment in Ago2 relative to the input. (H) The correlation between SNHG17 and miR-214-3p was analyzed in 90 paired OC samples ($n = 90$; $r = -0.291$, $p < 0.001$). $^{*}p < 0.05$; $^{**}p < 0.01$; $^{***}p < 0.001$.

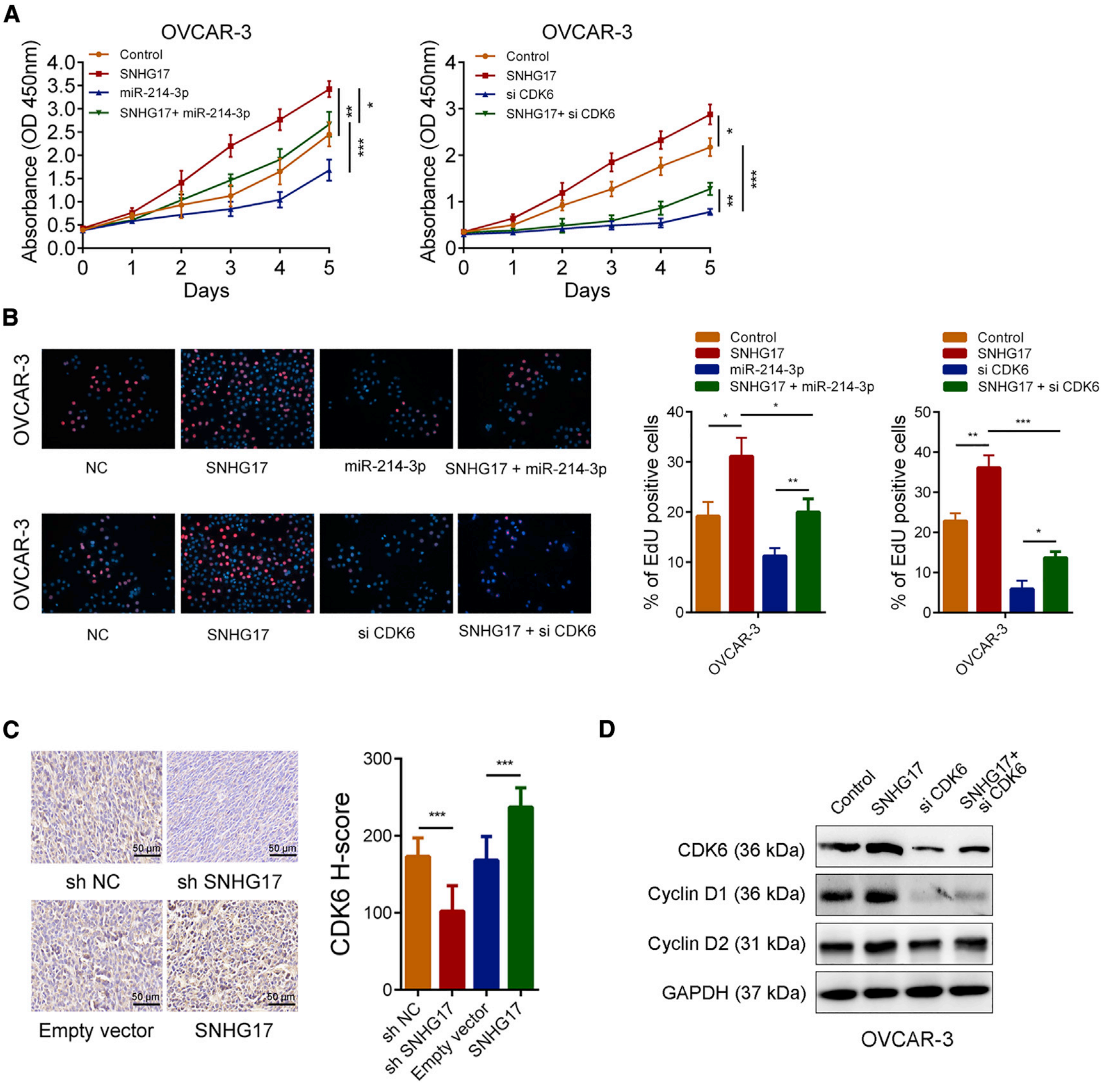

Figure 7. SNHG17 Promotes OC Cell Growth by Regulating CDK6 Expression

(A) The CCK-8 assays demonstrated that SNHG17 promoted OC cell growth, while miR-214-3p overexpression or CDK6 knockdown could abolish the growth promotion caused by SNHG17. (B) EdU assays showed that miR-214-3p overexpression or CDK6 knockdown abolished the increased proliferation rates of OVCAR-3 cells caused by SNHG17. (C) Left panel: representative images of the CDK6 immunostaining of the tumor samples from the different groups. Right panel: CDK6's H-score statistics result. (D) Expression of CDK6, cyclin D1, and cyclin D2 was detected by western blot in OVCAR-3 cells with the indicated treatment. *$p < 0.05$; **$p < 0.01$; ***$p < 0.001$.

pcDNA3.1-transfected OVCAR-3 cells were selected for 2 weeks using G418, and stable clones of shSNHG17 and shNC-transfected OVCAR-3 cells were selected for 2 weeks using puromycin. After selection, the expression level of SNHG17 was determined by qRT-PCR.

RNA FISH

FISH assays were performed using the Fluorescence *In Situ* Hybridization Kit (RiboBio, Guangzhou, China) according to the manufacturer's protocol. Cy3-labeled SNHG17 probes were designed and synthesized by RiboBio.

Subcellular Fractionation Location

The separation of the nuclear and cytosolic fractions was performed using the PARIS Kit (Invitrogen, Carlsbad, CA, USA) according to the manufacturer's instructions.

Luciferase Reporter Assay

For the SNHG17 promoter luciferase reporter assay, different fragment sequences containing the predicted STAT3 binding sites were synthesized and cloned into the pGL3-basic firefly luciferase reporter (GeneCreate, Wuhan, Hubei, China). The pRL-TK vector was used as a control. For microRNA target gene luciferase reporter assays, the

SNHG17 sequences containing the predicted miR-214-3p binding sites (Luc-SNHG17-WT) or containing mutations in the predicted microRNA binding sites (Luc-SNHG17-mt) were respectively synthesized and inserted into the pmirGLO luciferase vector (GeneCreate, Wuhan, Hubei, China). OVCAR-3 cells (1×10^5) were seeded in 24-well plates for 24 h. Mimics of miR-214-3p were cotransfected with 10 μg WT or mutated reporters. Twenty-four hours after transfection, a dual-luciferase reporter assay (Promega, Madison, WI, USA) was performed to measure the relative luciferase activity. The same procedure was used to assess the binding effect between CDK6 3′ UTR and miR-214-3p.

Xenograft Tumor Formation

The xenografted tumor model was established to assess the effect of SNHG17 *in vivo*. Briefly, 1×10^7 OVCAR-3 cells (shNC, shSNHG17, empty vector, and SNHG17 vector stably transfected) in 0.2 mL PBS were subcutaneously injected into BABL/c nude mice (20 per group). The tumor volumes were measured every 5 days, calculated with the following equation: $V = 0.5 \times$ (length $\times$ square width). One month later, the mice were sacrificed, and the tumors were surgically dissected for analysis.

Statistical Analysis

All statistical analyses were performed using SPSS v.20.0. The survival curves were calculated using the Kaplan-Meier method and analyzed using the log-rank test. For comparisons, one-way analyses of variance and two-tailed Student's t tests were performed as appropriate. $p < 0.05$ was considered statistically significant.

A complete description of the methods, including the CCK-8 assay and colony formation assay, the EdU incorporation assay, flow cytometry, RNA isolation and quantitative real-time PCR, protein extraction and western blot, IHC, GSEA, ChIP assay, and RIP assay are available in the Supplemental Materials and Methods.

SUPPLEMENTAL INFORMATION

Supplemental Information can be found online at https://doi.org/10.1016/j.omtn.2020.08.006.

AUTHOR CONTRIBUTIONS

X.P., Z.G., Y.Y., Y.C., S.Z., and M.P. conducted the experiments. X.P., Z.G., and Y.Y. participated in development of the methodology. Y.C. and M.P. assisted in data interpretation. Z.W., X.P., and Z.G. designed experiments and wrote the manuscript. All authors read and approved the final manuscript.

CONFLICTS OF INTEREST

The authors declare no competing interests.

ACKNOWLEDGMENTS

This work was financially supported by the Science International Cooperation Project of Jilin Province (20170414035GH). This work was also financially supported by the Department of Gynecologic Oncology of The First Hospital of Jilin University.

REFERENCES

1. Siegel, R.L., Miller, K.D., and Jemal, A. (2017). Cancer Statistics, 2017. CA Cancer J. Clin. *67*, 7–30.
2. Webb, P.M., and Jordan, S.J. (2017). Epidemiology of epithelial ovarian cancer. Best Pract. Res. Clin. Obstet. Gynaecol. *41*, 3–14.
3. Sankaranarayanan, R., and Ferlay, J. (2006). Worldwide burden of gynaecological cancer: the size of the problem. Best Pract. Res. Clin. Obstet. Gynaecol. *20*, 207–225.
4. Allemani, C., Weir, H.K., Carreira, H., Harewood, R., Spika, D., Wang, X.S., Bannon, F., Ahn, J.V., Johnson, C.J., Bonaventure, A., et al.; CONCORD Working Group (2015). Global surveillance of cancer survival 1995-2009: analysis of individual data for 25,676,887 patients from 279 population-based registries in 67 countries (CONCORD-2). Lancet *385*, 977–1010.
5. Matz, M., Coleman, M.P., Carreira, H., Salmerón, D., Chirlaque, M.D., and Allemani, C.; CONCORD Working Group (2017). Worldwide comparison of ovarian cancer survival: Histological group and stage at diagnosis (CONCORD-2). Gynecol. Oncol. *144*, 396–404.
6. Iyer, M.K., Niknafs, Y.S., Malik, R., Singhal, U., Sahu, A., Hosono, Y., Barrette, T.R., Prensner, J.R., Evans, J.R., Zhao, S., et al. (2015). The landscape of long noncoding RNAs in the human transcriptome. Nat. Genet. *47*, 199–208.
7. Batista, P.J., and Chang, H.Y. (2013). Long noncoding RNAs: cellular address codes in development and disease. Cell *152*, 1298–1307.
8. Gibb, E.A., Brown, C.J., and Lam, W.L. (2011). The functional role of long non-coding RNA in human carcinomas. Mol. Cancer *10*, 38.
9. Shi, X., Sun, M., Liu, H., Yao, Y., and Song, Y. (2013). Long non-coding RNAs: a new frontier in the study of human diseases. Cancer Lett. *339*, 159–166.
10. Gao, Y., Meng, H., Liu, S., Hu, J., Zhang, Y., Jiao, T., Liu, Y., Ou, J., Wang, D., Yao, L., et al. (2015). LncRNA-HOST2 regulates cell biological behaviors in epithelial ovarian cancer through a mechanism involving microRNA let-7b. Hum. Mol. Genet. *24*, 841–852.
11. Jin, Y., Feng, S.J., Qiu, S., Shao, N., and Zheng, J.H. (2017). LncRNA MALAT1 promotes proliferation and metastasis in epithelial ovarian cancer via the PI3K-AKT pathway. Eur. Rev. Med. Pharmacol. Sci. *21*, 3176–3184.
12. Liu, E., Liu, Z., and Zhou, Y. (2015). Carboplatin-docetaxel-induced activity against ovarian cancer is dependent on up-regulated lncRNA PVT1. Int. J. Clin. Exp. Pathol. *8*, 3803–3810.
13. Liang, H., Yu, T., Han, Y., Jiang, H., Wang, C., You, T., Zhao, X., Shan, H., Yang, R., Yang, L., et al. (2018). LncRNA PTAR promotes EMT and invasion-metastasis in serous ovarian cancer by competitively binding miR-101-3p to regulate ZEB1 expression. Mol. Cancer *17*, 119.
14. Ma, Z., Gu, S., Song, M., Yan, C., Hui, B., Ji, H., Wang, J., Zhang, J., Wang, K., and Zhao, Q. (2017). Long non-coding RNA SNHG17 is an unfavourable prognostic factor and promotes cell proliferation by epigenetically silencing P57 in colorectal cancer. Mol. Biosyst. *13*, 2350–2361.
15. Zhang, G., Xu, Y., Wang, S., Gong, Z., Zou, C., Zhang, H., Ma, G., Zhang, W., and Jiang, P. (2019). LncRNA SNHG17 promotes gastric cancer progression by epigenetically silencing of p15 and p57. J. Cell Physiol. *234*, 5163–5174.
16. Xu, T., Yan, S., Jiang, L., Yu, S., Lei, T., Yang, D., Lu, B., Wei, C., Zhang, E., and Wang, Z. (2019). Gene Amplification-Driven Long Noncoding RNA SNHG17 Regulates Cell Proliferation and Migration in Human Non-Small-Cell Lung Cancer. Mol. Ther. Nucleic Acids *17*, 405–413.
17. Khan, A., Fornes, O., Stigliani, A., Gheorghe, M., Castro-Mondragon, J.A., van der Lee, R., Bessy, A., Chèneby, J., Kulkarni, S.R., Tan, G., et al. (2018). JASPAR 2018: update of the open-access database of transcription factor binding profiles and its web framework. Nucleic Acids Res. *46* (D1), D260–D266.
18. Liu, W., and Wang, X. (2019). Prediction of functional microRNA targets by integrative modeling of microRNA binding and target expression data. Genome Biol. *20*, 18.
19. Wong, N., and Wang, X. (2015). miRDB: an online resource for microRNA target prediction and functional annotations. Nucleic Acids Res. *43*, D146–D152.
20. Du, Y., Wei, N., Hong, J., and Pan, W. (2020). Long non-coding RNASNHG17 promotes the progression of breast cancer by sponging miR-124-3p. Cancer Cell Int. *20*, 40.

21. Li, H., Li, T., Huang, D., and Zhang, P. (2020). Long noncoding RNA SNHG17 induced by YY1 facilitates the glioma progression through targeting miR-506-3p/CTNNB1 axis to activate Wnt/β-catenin signaling pathway. Cancer Cell Int. *20*, 29.

22. Kertesz, M., Iovino, N., Unnerstall, U., Gaul, U., and Segal, E. (2007). The role of site accessibility in microRNA target recognition. Nat. Genet. *39*, 1278–1284.

23. Vejnar, C.E., Blum, M., and Zdobnov, E.M. (2013). miRmap web: Comprehensive microRNA target prediction online. Nucleic Acids Res. *41*, W165–W168.

24. Agarwal, V., Bell, G.W., Nam, J.W., and Bartel, D.P. (2015). Predicting effective microRNA target sites in mammalian mRNAs. eLife *4*, e05005.

25. Reid, B.M., Permuth, J.B., and Sellers, T.A. (2017). Epidemiology of ovarian cancer: a review. Cancer Biol. Med. *14*, 9–32.

26. Wu, D.D., Chen, X., Sun, K.X., Wang, L.L., Chen, S., and Zhao, Y. (2017). Role of the lncRNA ABHD11-AS_1 in the tumorigenesis and progression of epithelial ovarian cancer through targeted regulation of RhoC. Mol. Cancer *16*, 138.

27. Wang, H., Fang, L., Jiang, J., Kuang, Y., Wang, B., Shang, X., Han, P., Li, Y., Liu, M., Zhang, Z., and Li, P. (2018). The cisplatin-induced lncRNA PANDAR dictates the chemoresistance of ovarian cancer via regulating SFRS2-mediated p53 phosphorylation. Cell Death Dis. *9*, 1103.

28. Martini, P., Paracchini, L., Caratti, G., Mello-Grand, M., Fruscio, R., Beltrame, L., Calura, E., Sales, G., Ravaggi, A., Bignotti, E., et al. (2017). lncRNAs as Novel Indicators of Patients' Prognosis in Stage I Epithelial Ovarian Cancer: A Retrospective and Multicentric Study. Clin. Cancer Res. *23*, 2356–2366.

29. Mohamadi, M., Ghaedi, H., Kazerouni, F., Erfanian Omidvar, M., Kalbasi, S., Shanaki, M., Miraalamy, G., and Rahimipour, A. (2019). Deregulation of long noncoding RNA SNHG17 and TTC28-AS1 is associated with type 2 diabetes mellitus. Scand. J. Clin. Lab. Invest. *79*, 519–523.

30. Chen, L.L., He, J., Qiu, X.T., Yu, J., and Wang, Z.M. (2019). The prognostic roles of long non-coding RNA SNHG17 in the patients with gastric cancer. Eur. Rev. Med. Pharmacol. Sci. *23*, 1063–1068.

31. Siveen, K.S., Sikka, S., Surana, R., Dai, X., Zhang, J., Kumar, A.P., Tan, B.K., Sethi, G., and Bishayee, A. (2014). Targeting the STAT3 signaling pathway in cancer: role of synthetic and natural inhibitors. Biochim. Biophys. Acta *1845*, 136–154.

32. Avalle, L., Pensa, S., Regis, G., Novelli, F., and Poli, V. (2012). STAT1 and STAT3 in tumorigenesis: A matter of balance. JAK-STAT *1*, 65–72.

33. Loh, C.Y., Arya, A., Naema, A.F., Wong, W.F., Sethi, G., and Looi, C.Y. (2019). Signal Transducer and Activator of Transcription (STATs) Proteins in Cancer and Inflammation: Functions and Therapeutic Implication. Front. Oncol. *9*, 48.

34. Vermeulen, K., Van Bockstaele, D.R., and Berneman, Z.N. (2003). The cell cycle: a review of regulation, deregulation and therapeutic targets in cancer. Cell Prolif. *36*, 131–149.

35. Tigan, A.S., Bellutti, F., Kollmann, K., Tebb, G., and Sexl, V. (2016). CDK6-a review of the past and a glimpse into the future: from cell-cycle control to transcriptional regulation. Oncogene *35*, 3083–3091.

36. Agirre, X., Vilas-Zornoza, A., Jiménez-Velasco, A., Martin-Subero, J.I., Cordeu, L., Gárate, L., San José-Eneriz, E., Abizanda, G., Rodríguez-Otero, P., Fortes, P., et al. (2009). Epigenetic silencing of the tumor suppressor microRNA Hsa-miR-124a regulates CDK6 expression and confers a poor prognosis in acute lymphoblastic leukemia. Cancer Res. *69*, 4443–4453.

37. Zhu, X., Li, Y., Shen, H., Li, H., Long, L., Hui, L., and Xu, W. (2013). miR-137 inhibits the proliferation of lung cancer cells by targeting Cdc42 and Cdk6. FEBS Lett. *587*, 73–81.

38. Xu, S., Huang, H., Li, N., Zhang, B., Jia, Y., Yang, Y., Yuan, Y., Xiong, X.D., Wang, D., Zheng, H.L., and Liu, X. (2016). MicroRNA-33 promotes the replicative senescence of mouse embryonic fibroblasts by suppressing CDK6. Biochem. Biophys. Res. Commun. *473*, 1064–1070.

OMTN, Volume *22*

Supplemental Information

STAT3-Induced lncRNA SNHG17 Exerts Oncogenic Effects on Ovarian Cancer through Regulating CDK6

Xuefeng Pan, Zhiheng Guo, Yanyan Chen, Shu Zheng, Min Peng, Yi Yang, and Zhenpeng Wang

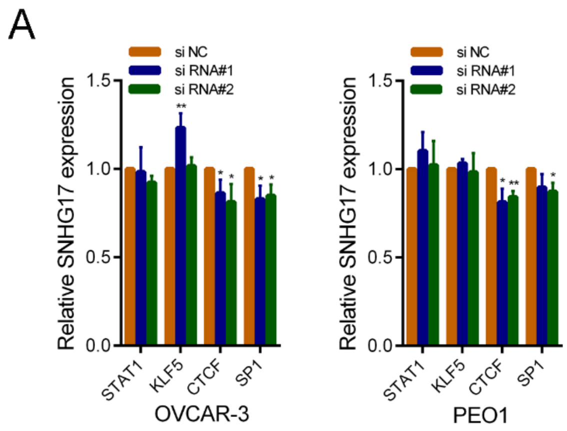

Figure S1. Explore potential transcription factors affecting SNHG17 transcription. **(A)** The SNHG17 expression was detected in OVCAR-3 and PEO1 cells transfected with STAT1, KLF5, CTCF or SP1 siRNAs. *P < 0.05 and **P < 0.01.

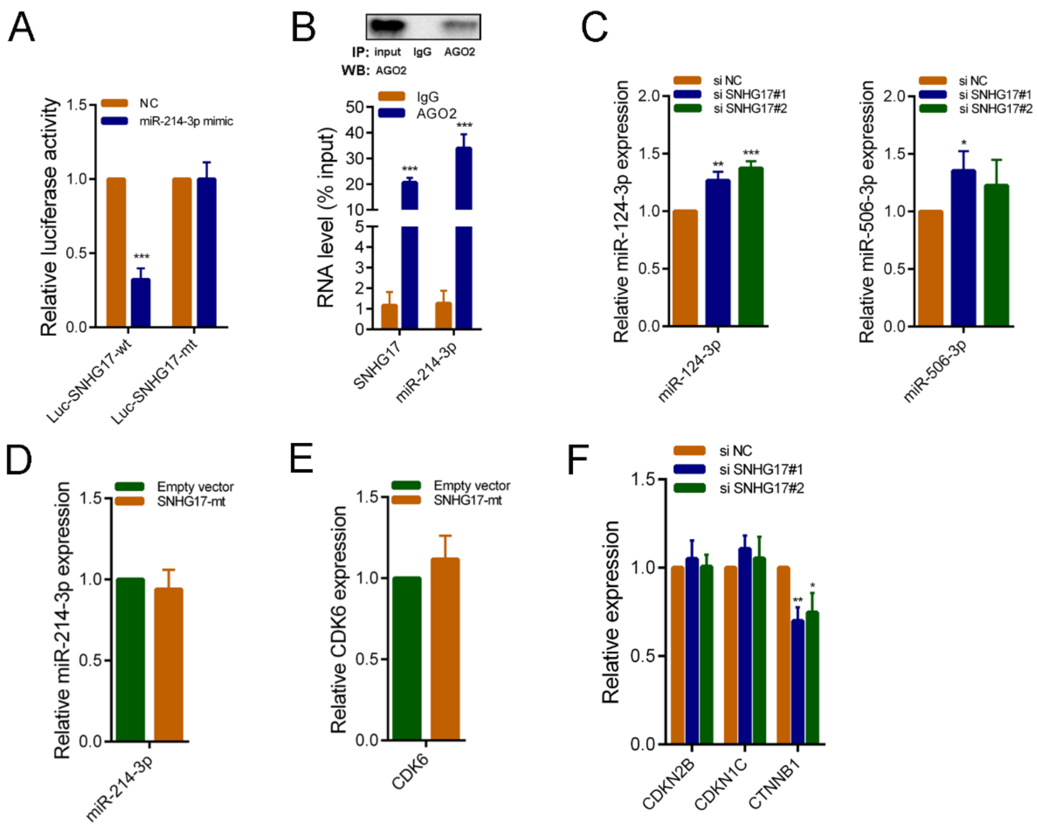

Figure S2. SNHG17 regulates CDK6 expression through sponging miR-214-3p in OC. (A) Dual-luciferase reporter assays of wild type (Luc-SNHG17-wt) and mutant type (Luc-SNHG17-mt) luciferase report vectors in PEO1 cells. **(B)** RIP assays with an anti-Ago2 antibody to assess endogenous Ago2 binding RNAs in PEO1 cells. **(C)** MiR-124-3p and miR-506-3p expression was detected in OVCAR-3 cells transfected with SNHG17 siRNAs. **(D)** MiR-214-3p expression was detected in OVCAR-3 cells transfected with SNHG17-mt vectors. **(D)** CDK6 expression was detected in OVCAR-3 cells transfected with SNHG17-mt vectors. **(E)** Expression of CDKN2B, CDKN1C and CTNNB1 was detected by qRT-PCR in OVCAR-3 cells after transfection with SNHG17 siRNAs. $^{*}P < 0.05$, $^{**}P < 0.01$ and $^{***}P < 0.001$.

Supplemental Methods

CCK-8 assay and colony formation assay

For CCK-8 assay, OVCAR-3 and PEO1 cells were seeded into 96-well plates at the density of 1×10^3 (cells/well), and the absorbance at 450nm was measured on days 1, 2, 3, 4 and 5 with 10 μl of CCK-8 solution treated. For cell colony formation assays, 24 hours after transfection, 500 OVCAR-3 or PEO1 cells were incubated in 6-well plates at 37˚C, 5% CO_2. Two weeks later, the cells were stained with crystal violet (0.2%) for 30 minutes and the colony numbers were counted.

5-Ethynyl-20-deoxyuridine (EdU) incorporation assay

OVCAR-3 and PEO1 cells were seeded at a density of 5×10^3 cells per well in 96-well plates and cultured overnight. The newly synthesized DNA of the cells was assessed by the EdU incorporation assay using a Cell-Light EdU DNA Cell Proliferation Kit (Ribobio, China), according to the manufacturer's instructions. The EdU incorporation rate was expressed as the ratio of EdU positive cells (red cells) to total Hoechst33342 positive cells (blue cells).

Flow cytometry

Flow cytometry cell apoptosis was analyzed using the Annexin V-FITC/ (PI) Apoptosis Detection Kit (BD, USA) according to the protocol. OVCAR-3 and PEO1 cells were firstly trypsinized and washed with cold PBS, then they were stained with FITC and PI and then analyzed using FACScan (BD, USA). For cell cycle analysis, OVCAR-3 and PEO1 cells were firstly trypsinized and washed with cold PBS, then they were fixed in 70% ethanol for 24 hours and stained with Propidium Iodide (PI) for 30 minutes. Finally, the cells were analyzed by FACScan flow cytometer (BD, USA). The cell apoptosis data and cell cycle data was analyzed by Flowjo software (Tree Star, USA).

RNA isolation and quantitative real-time PCR

Total RNA was extracted from tissues and cells using TRIzol reagent (Invitrogen, Carlsbad, CA, USA). Reverse transcription of lncRNA and mRNA was performed using a PrimeScriptTM RT Master Mix Kit (TaKaRa, Osaka, Japan). MicroRNA expression was detected by a Hairpin microRNA quantitation kit (Genepharma, China). Quantitative RT-PCR was performed using a standard protocol from the SYBR Green

PCR kit (Toyobo, Osaka, Japan). The following primer sequences were used for qRT-PCR: for SNHG17, TGCTTGTAAGGCAGGGTCTC (forward) and ACAGCCACTGAAAGCATGTG (reverse); for CDK6, CCAGATGGCTCTAACCTCAGT (forward) and AACTTCCACGAAAAAGAGGCTT (reverse); ; for CDKN2B (P15), ACTAGTGGAGAAGGTGCGACAG (forward) and GCTGCCCATCATCATGACCTG (reverse) ; for CDKN1C (P57), TGAACGCCGAGGACCAGAA (forward) and ACCGAGTCGCTGTCCACTT (reverse) ; for CTNNB1, CCATCTGTGCTCTTCGTCATCT (forward) and GGAATGGCACCCTGCTCAC (reverse); for GAPDH, ATGGGGAAGGTGAAGGTCGG (forward) and GACGGTGCCATGGAATTTGC (reverse).

Protein extraction and western blot

Total proteins were extracted from cultured cells using cell lysis buffer. Then, the protein samples were loaded onto 10% sodium dodecyl sulfate polyacrylamide gel electrophoresis. The membranes were blocked with 5 % non-fat milk in Tris-buffered saline and incubated with a specific primary antibody and a secondary antibody. Protein expression was detected by enhanced chemiluminescence kit. Antibodies used in western blot: anti-Cyclin D1(#55506, Cell Signaling Technology, USA), anti-Cyclin D2(#3741, Cell Signaling Technology, USA) anti-CDK6 (#13331, Cell Signaling Technology, USA) and anti-GAPDH (#5174, Cell Signaling Technology, USA).

Immunohistochemistry

IHC staining was performed using Dako Envision System (Dako, USA) according to the manufacturer's guidelines. The IHC-stained tissue sections were scored by two pathologists who were blinded to the clinical parameters, respectively. The percentage of immunostaining and the staining intensity (0, negative; 1+, weak; 2+, moderate; and 3+, strong) were recorded. An H-score was calculated using the following formula: [1 × (% cells 1+) + 2 × (% cells 2+) + 3 × (% cells 3+)] × 100. The maximum H-score would be 300, corresponding to 100% of cells with strong intensity. Antibodies used in

IHC: anti-Ki-67 (#9449, Cell Signaling Technology, USA) anti-CDK6 (#13331, Cell Signaling Technology, USA).

Gene set enrichment analysis

Gene set enrichment analysis (GSEA) was used to explore the pathways and gene sets associated with SNHG17 in ovarian cancer. Gene expression profiles of 370 ovarian cancer samples were downloaded from TCGA database. According to the SNHG17 expression level, the samples were grouped as the high SNHG17 group and low SNHG17 group respectively. GSEA v3.0 was used to determine whether the members of the gene sets from the MSigDB database were randomly distributed at the top or bottom of the ranking. If most members of a gene set were positively or negatively related to SNHG17, the set was associated with SNHG17 expression.

Chromatin immunoprecipitation assay

ChIP assays were performed using the ChIP Assay Kit (Beyotime, China) according to the manual with slight modifications. OVCAR-3 and PEO1 cells were cross-linked with 1% formaldehyde solution for 10 min at room temperature and quenched with 125 mM glycine. DNA fragments ranging from 200 to 500 bp were yielded via sonication. Then the lysates were immunoprecipitated with anti-STAT3 (#12640, Cell Signaling Technology, USA) or normal rabbit IgG antibody. Immunoprecipitated DNAs were analyzed by qRT-PCR and ChIP primers for the promoter region of SNHG17 are: GATTTGTGGGCCATGAGTCC (forward) and GCGTGAGAAGATGAGCCAAG (reverse).

RNA pull-down assay

SNHG17 template DNA was transcribed in vitro with Biotin RNA Labeling Mix and T7 RNA polymerase (Roche, Switzerland) and purified with a RNeasy Mini Kit (Qiagen, USA) according to the manufacturer's instructions. RNA-bound beads were incubated with total cell lysates of OVCAR-3, and eluted RNA was purified and assessed by qRT-PCR.

RNA immunoprecipitation assay

The RIP assay was performed using the Magna RNA immunoprecipitation Kit (Millipore, USA) following the the manual. Briefly, OC cells were lysed in RIP lysis

buffer. Magnetic beads were pre-incubated with anti-AGO2 (#ab32381, Abcam, USA) or IgG antibody for 30 minutes at room temperature and the cell lysates was immunoprecipitated with beads for 6 hours at 4°C. After that, RNA was purified and detected by qRT-PCR.
